# A Review of PRESAGE Radiochromic Polymer and the Compositions for Application in Radiotherapy Dosimetry

**DOI:** 10.3390/polym14142887

**Published:** 2022-07-16

**Authors:** Muhammad Zamir Mohyedin, Hafiz Mohd Zin, Mohd Zulfadli Adenan, Ahmad Taufek Abdul Rahman

**Affiliations:** 1 School of Physics and Material Studies, Faculty of Applied Sciences, Universiti Teknologi MARA, Shah Alam 40450, Selangor, Malaysia; zamirmohyedin@gmail.com; 2 Centre of Astrophysics & Applied Radiation, Institute of Science, Universiti Teknologi MARA, Shah Alam 40450, Selangor, Malaysia; 3 Advanced Medical & Dental Institute, Universiti Sains Malaysia, Bertam, Kepala Batas 13700, Penang, Malaysia; hafiz.zin@usm.my; 4 Centre of Medical Imaging, Faculty of Health Sciences, Universiti Teknologi MARA, Cawangan Selangor Campus of Puncak Alam, Puncak Alam 42300, Selangor, Malaysia; mohdzulfadli@uitm.edu.my

**Keywords:** radiation dosimeter, three-dimensional dosimetry, polymer dosimeter, PRESAGE, radiotherapy

## Abstract

Recent advances in radiotherapy technology and techniques have allowed a highly conformal radiation to be delivered to the tumour target inside the body for cancer treatment. A three-dimensional (3D) dosimetry system is required to verify the accuracy of the complex treatment delivery. A 3D dosimeter based on the radiochromic response of a polymer towards ionising radiation has been introduced as the PRESAGE dosimeter. The polyurethane dosimeter matrix is combined with a leuco-dye and a free radical initiator, whose colour changes in proportion to the radiation dose. In the previous decade, PRESAGE gained improvement and enhancement as a 3D dosimeter. Notably, PRESAGE overcomes the limitations of its predecessors, the Fricke gel and the polymer gel dosimeters, which are challenging to fabricate and read out, sensitive to oxygen, and sensitive to diffusion. This article aims to review the characteristics of the radiochromic dosimeter and its clinical applications. The formulation of PRESAGE shows a delicate balance between the number of radical initiators, metal compounds, and catalysts to achieve stability, optimal sensitivity, and water equivalency. The applications of PRESAGE in advanced radiotherapy treatment verifications are also discussed.

## 1. Introduction

**Figure 1:**
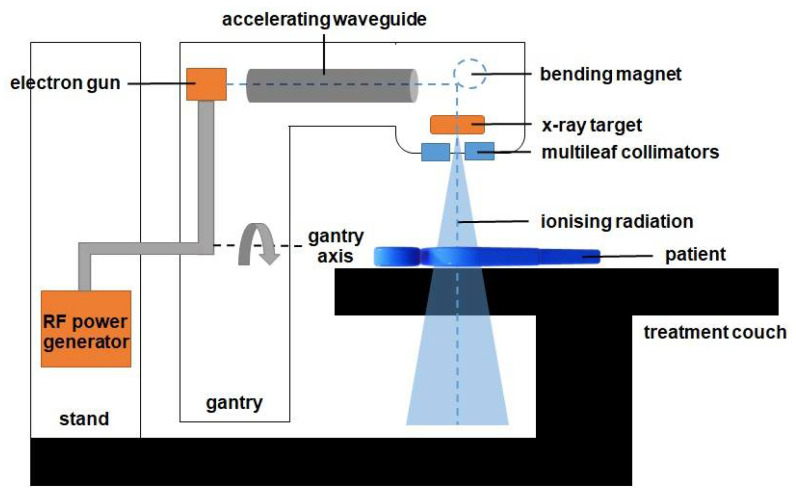
The diagram of components in the linac. The gantry is rotated to deliver the radiation from various angles to produce a 3D volumetric dose inside the patient. The beam is shaped using a set of multileaf collimators and delivered externally to the patient to kill the cancer cells inside the body.

Cancer is one of the critical health issues worldwide [1]. Radiotherapy remains an important curative technique in cancer treatment. Approximately half of all cancer patients obtained radiotherapy as a part of their treatment regime [2]. The treatment may also be combined with other treatment modalities, which include chemotherapy or surgery [3,4,5]. Radiotherapy involves the delivery of a radiation dose to the tumour while limiting the dose to the surrounding healthy tissues during treatment. Typically, the radiation dose is delivered externally from a linear accelerator (linac) or internally in certain types of cancers, using radioactive sources. Figure 1 shows a schematic diagram of a linac that produces a highly focused ionising radiation in the patient.

The ionising radiation is a high-energy X-ray produced by the X-ray targets, as shown in Figure 1. A wave guide is used to accelerate electrons in a part of the accelerator to allow these electrons to collide with the heavy metal X-ray target to produce the X-rays. These high-energy X-rays are shaped by a multileaf collimator system that is incorporated into the head of the machine to conform to the shape of the patient’s tumour. The beam can be rotated around the patient from many angles by rotating the gantry to direct the collimated beam to the patient’s tumour. The patient lies on a moveable treatment couch and setup, which is based on a laser positioning system to make sure that the patient is in the proper position. The treatment couch can move in many directions, including up, down, right, left, in, and out.

The radiotherapy technique has shown significant progress over the years following the advancement in linac technology. A significant development occurred in the radiation delivery techniques from 3D Conformal Radiotherapy (3D-CRT), through various Intensity Modulated Radiation Therapy (IMRT) approaches, such as Volumetric Modulated Arc Therapy (VMAT), Stereotactic Ablative Radiation Therapy (SABR), and Stereotactic Body Radiation Therapy (SBRT), to the implementation of Image-Guided Radiation Therapy (IGRT) upon providing IMRT treatment [6,7,8,9,10,11]. In addition, brachytherapy treatment has also become more conformal with the incorporation of an image guidance system [12,13]. These new techniques utilise a treatment planning system that is capable of calculating radiation doses in the heterogeneous patient through the inverse planning method [14]. In radiotherapy treatment planning, patient computed tomography (CT) images are acquired to establish the target volume and allow computation of the radiation dose based on radiation interaction modelling in the tissues [15]. The approaches enable the reduction of the dose to the surrounding normal tissue, thereby reducing radiation toxicity, whilst maximising the dose to the cancer target.

The modern approaches of radiotherapy produce three-dimensional (3D) dose distribution that requires a reliable dosimetry system to verify the administration of the radiation dose planned [16]. The radiation dose delivered to the target must be within a minimum of 95% accuracy and a tolerance of lower than 5% deviation to achieve the treatment intent [17]. The primary elements of the dosimetry system include a dosimeter and its reader. An ideal system should have high precision, outstanding accuracy, and good spatial resolution and linearity. An ideal dosimeter should also provide a dose measurement that is not dependent on the direction, dose rate, and energy of the radiation. Tissue equivalence is another important characteristic of a dosimeter as it reflects its capability to quantify the dose absorbed in water, which is important when considering that the human body consists mainly of water [18].

A few reviews on the development of the radiochromic polymer dosimeter, known as PRESAGE, have been conducted, such as those by Jordan in 2010, Schreiner in 2015, and Khezerloo et al. in 2017 [16,19,20]. In this work, we attempted to extensively review the development of PRESAGE from its fabrication, radiological properties, sensitivity, linearity, stability, reusability, reproducibility, dose-rate dependency, and energy dependency and its readout modalities and clinical applications. We also discussed in detail how the compounds in the PRESAGE, such as the metal compounds and various elements in the radical initiator, have an impact on its dosimetric ability. The recent development of a reusable PRESAGE was also included, looking in particular at the PRESAGE’s capability to reproduce the same dose response. We also reviewed the dose rate and energy dependency of PRESAGE at uncommon variables, such as an extremely low and high dose region. Finally, we discussed the application of PRESAGE as a dosimetry system for radiotherapy treatment.

### 1.1. Radiotherapy Dosimetry

Currently, there are many types of dosimeters for radiotherapy, including the ionisation chamber, films, the thermoluminescent dosimeter (TLD), and the array dosimeter [16,21,22,23]. The ionisation chamber is a gas-filled ionisation chamber, which comprises an electrode surrounded by a conductive wall that collects the charges generated from radiation interaction in the gas [24]. An electrometer quantifies the charges from the ion pairs produced within the gas. The film dosimeter consists of a radiosensitive thin layer, which darkens upon irradiation due to the polymerisation in a radiochromic dosimeter and emulsion in a radiographic dosimeter [25,26]. The film dosimeter can be read using a film scanner, transmission densitometers, or spectrophotometers [27]. Following that, the luminescence dosimeter emits luminescence upon irradiation, which can be read out thermally or optically. The TLD is stimulated by heat, while the optically stimulated luminescent dosimeter (OSLD) is stimulated by light [28]. In a semiconductor detector, irradiation causes the semiconductor to produce electron-hole pairs that are proportional to the radiation dose [29,30]. As a result, the dose absorbed by the semiconductor dosimeter can be read instantly [31].

The radiation dose delivery is distributed to the patient volume, making a 3D dosimeter an ideal detector that is capable of measuring the 3D dose distribution. In most cases, the commonly used detectors are one-dimensional (1D) or two-dimensional (2D). The IMRT treatment, for example, involves the complex movement of the gantry and multileaf collimator (MLC) to distribute a conformal radiation dose to the target. A challenge may be present in the 1D or 2D dosimeter to detect the whole volumetric dose distribution in the IMRT treatment. The dose distribution from the treatment can be effectively measured using a 3D dosimeter, given that the IMRT produces volumetric dynamic dose distribution. Some of the dosimetry systems can be designed to provide 3D dose distribution by arranging multiple detectors in a 2D or 3D phantom array. Several commercial 3D array detectors are designed to offer sensitive and convenient IMRT delivery verification [32,33,34]. The IMRT treatment is verified by measuring the delivered dose and comparing it with the prescribed dose. The dose measurement is conducted using a phantom in conjunction with a dosimeter, which is usually 1D or 2D. Although this method has been clinically practical for a patient’s specific 3D dose verification, it normally creates sparse 3D data of dose distribution. Therefore, an improved dosimetry system is important for precise 3D dose distribution verification [35]. A 3D dosimetry technique could be benchmarked using the Resolution-Time-Accuracy-Precision (RTAP) effectiveness criteria to determine whether it is a true 3D dosimeter [36,37]. Moreover, RTAP states that a dosimeter should be able to perform 3D dose measurement with 1 mm isotropic spatial resolution in a duration shorter than hour. The 3D dose should also show an accuracy of 3% and a precision of 1%.

### 1.2. Radiochromic Dosimeters

Currently, the chemical radiation dosimetry is the only actual 3D dosimeter with the characteristics of high-resolution isotropic measurement. The dose measured from the chemical 3D dosimeter is quantified upon the effect of the radiation-induced chemical changes in a material volume [38,39,40]. The 3D chemical dosimeter comprises three primary groups, namely Fricke gel, polymer gel, and radiochromic polymer. The emergence of the 3D chemical dosimeter took place in 1950 through the changes of colour recorded by Folin upon the exposure of phenol to the ionising radiation [41]. In 1958, monomer polymerisation was established as a dosimetry process whereby polymerisation occurs upon irradiation [42]. Notably, the gel dosimeter exhibits outstanding performance in measuring sophisticated 3D dose distribution, and it possesses tissue equivalency, strong spatial resolution, independence of radiation direction, and dose integration capability during the treatment [43,44].

#### 1.2.1. Fricke Gel Dosimeter

The Fricke dosimeter is a gel dosimeter based on ferrous ions (Fe^2+^) in gelatine matrix, which was initially examined in 1985. The ferrous ions in the gel are transformed into ferric ions (Fe^3+^), which create a chemical change upon irradiation [45]. A dosage map could be described through the use of magnetic resonance imaging (MRI) to conduct a measurement of the rate of the proton spin-lattice relaxation of the water molecules. Subsequently, a notable difference is acquired between the ferrous ions and the ferric ions as a result of their contrast in terms of magnetic moments [46]. In comparison to other gel dosimeters, the main advantages of the Fricke dosimeter include its easier preparation and high reproducibility [47,48]. However, the major drawback of the early Fricke dosimeter is the diffusion that occurs over time, resulting in the blurring of the dosage map and the spatial information [49,50]. Additionally, the radiation detection sensitivity becomes weaker during the increase in the linear energy transfer (LET), which hinders the absolute dose evaluation [50,51]. However, recently, low-diffusion Fricke gel has been developed, such as PVA-GTA Xylenol Orange Fricke gel [52,53]. Fricke gel has been improved into the MRI-based nanocomposite Fricke gel (NC-FG), which is free from the diffusion and has LET independence due to the incorporation of 1% (*w*/*w*) clay nanoparticles [50].

#### 1.2.2. Polymer Gel Dosimeter

The polymer gel dosimeter consists of five chemical ingredients, namely gelatine, water, catalyser, oxygen scavenger, and monomer [40]. The first investigation of polymer gel dosimeter was conducted in 1958 by Alexander et al., to examine, in particular, the effects of the ionised radiation on polymer gel [54]. Upon the absorption of the ionising radiation by the polymer gel, the gel polymerised and the cross-linking of monomers took place, following the generation of free radicals [55,56]. The radiation-sensitive element of the polymer gel is acrylamide or methacrylic monomers [20]. In line with the Fricke gel, the dosage map of the polymer gel can be read out using MRI [41,57,58]. The change in the water molecule excitability through polymerisation changes the relaxation time of its proton spin lattice. The radiation also influences the mass density, elasticity, and opacity of the polymer gel, enabling the use of optical scanning, X-ray computed tomography, and ultrasonography as a dosage map readout [58,59,60].

As observed in the Fricke gel, no diffusion issue is present in polymer gels. However, the first production of polymer gel dosimeters, which are commercially known as BANANA gel (bis, acrylamide, nitrous oxide, and agarose) and BANG (bis, acrylamide, nitrogen and gelatine), is faced with challenges in fabrication due to the strong sensitivity to oxygen [61]. Moreover, the condition for oxygen control upon fabrication, imaging, and irradiating has impeded the simplicity of the use and stability of the polymer gel dosimeters. Oxygen also restricts polymerisation, bearing in mind that oxygen scavenges the free radicals [62]. Consequently, the sensitivity to oxygen leads to a vague radiation response from the polymer gel [18].

To manage the issue, the polymer gel was modified by Fong et al. with the creation of a normoxic gel, commercially known as MAGIC, which is composed of methacrylic acid, ascorbic acid, gelatine, and copper sulphate [63]. The purpose of these new ingredients is to eliminate the gel sensitivity to oxygen whilst still keeping the same properties of the previous gel. The ascorbic acid is an oxygen scavenger that helps to remove or reduce the oxygen inhibition [64]. Additionally, the presence of an oxygen scavenger has increased the dose response at low scavenger levels [65]. Furthermore, the oxygen scavengers have made the oxygen itself initiate free radicals that are responsible for initiating polymerisation. This new polymer gel owes its outstanding advancement to the fabrication of normoxic gels that can be made without the need to control the oxygen.

In 2002, Deene et al. changed the composition of MAGIC gel and suggested a few formulations [66]. Following the investigation conducted on another oxygen scavenger, it was found that tetrakis (hydroxymethyl) phosphonium chloride (THPC) possessed high potency in scavenging oxygen, with the effectiveness level being directly proportional to its concentration. Therefore, the concentration of oxygen scavenger can be decreased to increase the dose sensitivity of the gel. The dose sensitivity could increase by the factor of 3 when the oxygen scavengers were at their minimum compared to the gel of higher oxygen scavenger concentration [67].

The production of another commercial polymer gel, known as MAGAT, has replaced the copper sulphate and ascorbic acid with THPC [66]. This was followed by the production of other forms of normoxic gel, including MAGAS (methacrylic acid, gelatine, and ascorbic acid). Despite the improvement in polymer gel as a result of the presence of THPC, many issues were found in methacrylic acid-based normoxic gels during post-irradiation, given the temporal and spatial instabilities resulting from strong acidity and long-lived radicals [68]. Furthermore, methacrylic acid also strengthened the background or noise of the response curve [63]. The methacrylic acid-based normoxic gels were replaced with acrylamide-based normoxic gels, which are commercially known as PAGAT, that are composed of polyacrylamide gel and THPC. This replacement was made as a solution to issues related to the use of methacrylic acid. However, the high toxicity of acrylamide demonstrates teratogenic and mutagenic risks [67,69]. Besides exhibiting lower sensitivity as compared to the methacrylic-based normoxic gels, acrylamide-based normoxic gels also have lower sensitivity than the traditional acrylamide polymer gels such as PAG and BANG [40,70].

#### 1.2.3. Radiochromic Polymer Dosimeter

**Figure 2:**
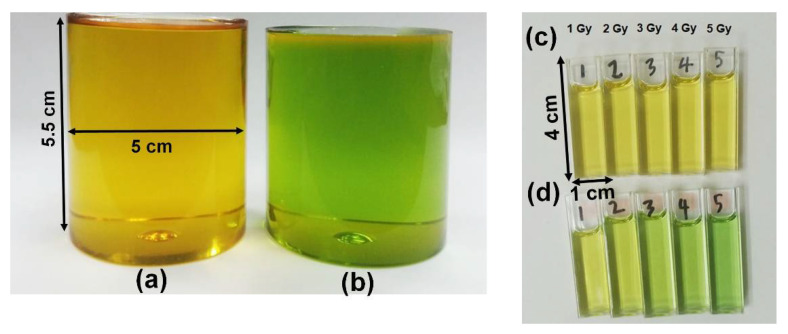
(**a**) The original colour of our batch of PRESAGE is yellow. (**b**) The PRESAGE changes colour to green upon irradiation. (**c**) The original state of PRESAGE in cuvette. (**d**) The colour becomes greener as the dose is increased.

PRESAGE is a commercial radiochromic polymer dosimeter that was introduced by John Adamovics [71]. It exhibits several strong advantages compared to other polymer gel dosimeters; it is insensitive to oxygen contamination and can be fabricated with any desired shape without any vessels; it does not exhibit diffusion and has high stability and higher optical evaluation [19,20,35,72]. Moreover, the presence of oxygen can increase the dosimeter sensitivity upon fabrication [73]. It is also a tissue equivalent in the range of megavoltage energies, which is suitable for radiotherapy. Additionally, the dosimeter response to radiation is not dependent on the dose rate and a wide range of energy [20]. The colour change in PRESAGE upon irradiation is illustrated in Figure 2.

Several factors should be considered for the fabrication of an optimal PRESAGE; these include radiation sensitivity, stability during fabrication and post-irradiation, optical transparency, and tissue equivalency [74,75,76]. The PRESAGE has been reformulated several times to achieve optimal sensitivity, stability, and water equivalency. The incorporation of metal compounds and different types of radical initiators and oxygen have simultaneously affected the three properties. Thus, careful formulation should be made to produce an optimised PRESAGE. An excellent dosimeter should be able to produce a linear response. A low radiation dose produces a lower response to the PRESAGE. The response becomes stronger as the delivered dose increases. The PRESAGE is capable of producing good linearity with a correlation coefficient of higher than 0.99. Over the course of the PRESAGE reformulation, the linearity of the PRESAGE showed almost no deterioration.

## 2. The Components of PRESAGE

The main material of PRESAGE is polyurethane, which consists of 61% carbon, 20% oxygen, 10% nitrogen, and 9% hydrogen. It has an effective atomic number of 6.6 and a density of 1.05 g/cm^3^ [77,78]. Polyurethane has a clear solid form and can polymerise at low temperatures, which is crucial to ensuring the reduction in the unwanted thermal oxidation reactions that amplify the background radiochromic reaction [71]. The radiochromic part of PRESAGE is made up of leucomalachite green (LMG) dye and a halocarbon free radical initiator. Notably, LMG shows maximum absorbance at 633 nm. The free radicals created from halocarbon radiolysis and oxidisation during radiation interaction change the LMG into malachite green (MG) [61,79]. The change of optical density has developed the dosimeter into a colour agent that can be read out.

PRESAGE fabrication comprises two steps, which are the fabrication of the polymer and the addition of the leuco-dye [20,77,80]. The first step requires the formation of prepolymer, which involves a reaction of a molar equivalent of polyol with two molar equivalents of diisocyanate. Polyol is an organic compound carrying multiple hydroxyl groups (OH), whereas the diisocyanate is an organic compound of two isocyanate groups. A non-reactive prepolymer that can be stored at room temperature is created due to the reaction of these compounds. The chemical reaction of the first step can be described as follows:

HO − R1 − OH (polyol) + 2OCN − R2 − NCO (diisocynate) → OCN − R2 − [−NH − C(=O) − O − R1 − O − C(=O) − NH − R2−] n − NCO (prepolymer, part A)(1)

The second step is where the leuco-dye, free radical initiator, polyol, and a catalyst are combined. The product from this combination is integrated with the prepolymer created from the first step to obtain a homogenous mixture. The mixture is placed in a suitable mould and maintained under the pressure of 60 psi at the optimum temperature to reduce outgassing. The chemical response of the second phase can be described as follows:

Part A + HO − R3 − OH (Part B) + leuco dye + free radical initiator + catalyst → [(C(=O) − NH − R2 − NH − C(=O) − O − R1 − O − C(=O) − NH − R2) n − NH − C(=O) − O − R3 − O] − m + leuco dye + free radical initiator + catalyst

**Figure 3:**
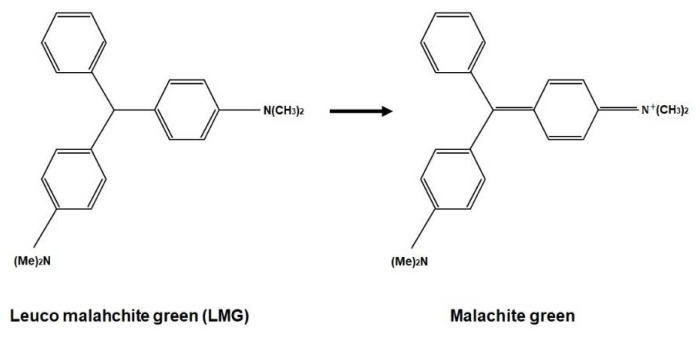
The chemical formula of radiochromic response.

Besides polyurethane, other base materials have been utilised to fabricate PRESAGE, such as epoxies, polyesters, acrylics, polycarbonates, polystyrene, and polyvinyl chloride (PVC). However, polyurethane shows several advantages compared to other materials. For instance, the effective atomic number of PVC does not hold the same value as the tissue. Additionally, the heat produced during the polymerisation of polycarbonates, polystyrene, acrylics, and polyesters at >100 °C can degrade the leuco-dye in PRESAGE. Epoxy, on the other hand, has low radiation sensitivity [77]. Figure 3 presents the chemical formula of the radiochromic response due to the irradiation of PRESAGE.

A number of other organic compounds can be used as a leuco-dye; these include crystal violet lactone, green diaminofluoran, orange diaminofluoran, black fluoran, and leucomalachite green (LMG). The wide use of LMG as a leuco-dye in the fabrication of PRESAGE is due to its higher sensitivity and high reactivity to high-energy radiation as compared with the other organic compounds. Furthermore, LMG shows the highest visible absorbance at 633 nm, while the green diaminofluoran, orange aminofluoran, and black fluoran show the lowest sensitivity and response [77]. As for the free radical initiator, several materials can be employed, such as organic peroxides, carbon tetrachloride, halogenated carbons, halogenated hydrocarbons, azo compounds, sulphur components, and carbonyl [71,78,79,80,81]. Halogenated carbons (or halocarbons), such as methylene chloride and chloroform, can trigger oxidation of the leuco-dye in the water system [78,82]. Halocarbons are the organic compounds that have halogens such as iodine (I), chlorine (Cl), or bromine (Br) covalently bonded with one or more carbons.

Among the closest formulations of PRESAGE to water are those that employ methoxy-LMG as a new LMG derivative. The PRESAGE has an effective atomic number of 7.46, which holds a 0.54% difference from the effective atomic number of water (7.42) in the kilovoltage energy range [83]. In the megavoltage energy range, the PRESAGE has an effective atomic number of 7.69, with 3.57% difference from the effective atomic number of water [84].

## 3. Radiological Properties of PRESAGE

Extensive research has been conducted on the ionising radiation interaction probability in materials of diverse effective atomic number and density. Ionising radiation interacts with a material and deposit energy as it crosses along its path. High-energy radiation such as X-rays and gamma rays transfers most of its energy to secondary electrons that are generated by Compton, a photoelectric and pair production effect [85,86]. The interaction probability is heavily dependent on mass density (ρ), atomic number, and electron density ($\rho_{e}$). In addition, Compton scattering does not depend on the atomic number of the absorbing material to occur because the Compton scattering process only requires free electrons. Thus, it relies on electrons per gram of the material ($\rho_{e}/\rho$) [87].

In PRESAGE, the carbon consisted of over 60% elemental components. Compared to the oxygen that amounted to over 80% in water in low photon energies, carbon shows a lower attenuation coefficient [88]. In the kilovoltage energy range (10 kV–100 kV), where the photoelectric effect was prevalent, the higher energy absorption and the mass attenuation coefficient of PRESAGE were higher than water. However, due to the significant proportion of carbon, which has a low atomic number, the stopping power of PRESAGE is weaker than water, which has a high proportion of oxygen, which has a high atomic number.

### 3.1. The Role of Effective Atomic Number of Elements

Despite the major formulation of PRESAGE, which has a higher effective atomic number than water, it can be considered to have water equivalency in a good approximation at a higher energy range as long as the ratio of the electron density per the density of the material ($\rho_{e}/\rho$) remains closed to the water [72]. PRESAGE that has $\rho_{e}/\rho$ of 3.28 e/kg shows almost same photon probability interactions as water that has $\rho_{e}/\rho$ of 3.34 e/kg, which is only a less than 5% difference at an energy range above 300 keV to 30 MeV. However, due to the high effective atomic number, the photon interaction probability of the PRESAGE is not same as that of the water at the lower energy range, with the large difference of 81%. This indicates that the PRESAGE in the study is not suitable for low-dose dosimetry [72,89]. For the usage of higher radiation energy, the electron density and the mass density should be taken into account because of the Compton scattering dominancy at that range of energies. PRESAGE has made an improvement known as Formulation A to reduce the effective atomic number by adding a small percentage of sulphur and reducing the percentage of bromine (Br). The reduction by even a small percentage of Br decreased the effective atomic number of PRESAGE significantly (Z_eff_ = 7.56), due to the high atomic number of the Br.

According to a study, the prominent difference in the photoelectric absorption between the PRESAGE and water is 40%, due to the reduction of Br, which is a reduction by 41% when compared with the original PRESAGE [84,89]. Furthermore, the Compton scattering at the energy range of 2 MeV to 20 MeV of PRESAGE has a difference of 3% compared to water. For a pair production cross-section, PRESAGE has a maximum difference of 9% when compared with the water [84]. A study shows that PRESAGE has a percentage difference within 2% in photon absorption as compared with water over an energy range of 10 keV to 10 MeV, due to the addition of DBTDL, which was able to modify the effective atomic number of PRESAGE [90].

### 3.2. The Effect of Metal Compounds

The addition of metal compounds also has an effect on the photon interaction probability in PRESAGE. The increased percentage of metal compound in the PRESAGE would increase the effective atomic number. The increment is due to the high atomic number of metal atoms in the compound. A study shows that the PRESAGE that is incorporated with 3 mM of bismuth neodecanoate (Bi Neo) has a higher effective atomic number when compared with the PRESAGE that is incorporated with 3 mM of zinc octoate (Zn Oct), due to the high atomic number of Bi in the Bi Neo. Therefore, the photoelectric interaction probability of PRESAGE + Bi Neo is higher than PRESAGE + Zn Oct. Nevertheless, all the PRESAGE compositions used in the study do not have water equivalency at low energy ranges due to the higher effective atomic number than water. However, at high energy ranges, all the PRESAGEs have water equivalency due to the insignificant change of material density and electron density, especially at small concentration of the metal compounds [91]. This indicates that the delicate incorporation of metal compounds, even at low concentration, has to be considered for low energy ranges, which is very important for low-dose radiotherapy. In addition, the study also shows a negligible difference for predominant Compton scattering energy ranges when low concentrations of metal compounds are included in the PRESAGE composition.

A PRESAGE that is known as a metal optimised dosimeter (MOD), fabricated by Alqathami et al., illustrates the closest water equivalency for both the low- and the high-energy ranges [92]. It has an effective atomic number of 7.416, which is only a 0.013% difference from water. In addition, the photoelectric absorption cross-section for the PRESAGE (MOD) shows a less than 18% variation when compared with water. As compared with the previous study, the PRESAGE (MOD) has reduced the deviation from water by 22%. This improvement was attributed to an extremely small concentration of metal compounds in the formulation (~0.01 wt.%). Following that, the Br and Cl were reduced, while S was removed from the composition. The Compton scattering cross-section demonstrated an extremely small deviation due to the low physical density. The available photon cross-section of PRESAGE is illustrated in Table 1.

### 3.3. The Effect of Radical Initiator

**Figure 4:**
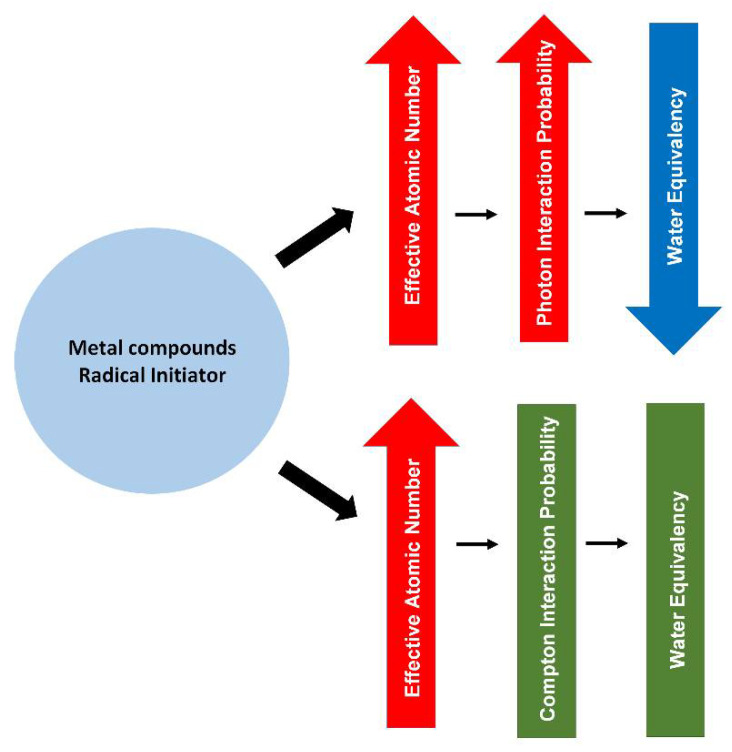
A diagram summarising the effect of metal compounds and radical initiator on the radiological properties of PRESAGE.

Among the halocarbons, iodoform demonstrated higher density (4.01 g/cm^3^) in comparison to the bromoform and chloroform, with the densities of 2.89 g/cm^3^ and 1.49 g/cm^3^, respectively. Nevertheless, PRESAGE that uses iodoform at 100 mM as a radical initiator shows a minor difference (<2.5%) in the Compton scattering cross-section, in comparison to water at the range of energy from 1 MeV to 20 MeV. Similarly, PRESAGE that employs iodoform shows a minor variation (<4%) for the pair production cross-section in comparison to water. In addition, due to the high atomic number of iodine (Z = 53), PRESAGE (iodoform 100 mM) has an effective atomic number of 16.03 [93]. This strongly indicates that the effective atomic number plays an insignificant role in water equivalency at megavoltage energies. However, PRESAGE (iodoform 100 mM) possesses a high deviation of more than 96% for photoelectric cross-section when compared with water. The most recent study, which used bromine-based RI PRESAGE, shows the small difference of 8% in the photoelectric absorption between the PRESAGE and water. This is due to the small fractional weight of Br in the composition. Increasing the proportion of Br has increased the percentage difference by more than 50%, due to the increment of the effective atomic number [94]. Figure 4 shows a simple diagram that summarises the effects of the metal compounds and the radical initiator on the radiological properties of PRESAGE.

## 4. Sensitivity and Linearity of PRESAGE

**Figure 5:**
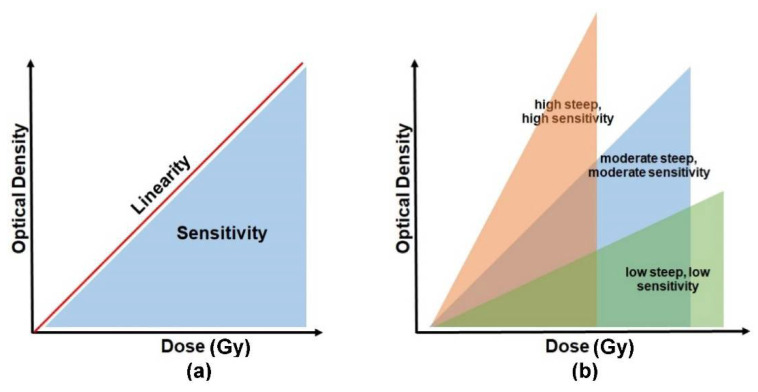
The basic concept to interpret the sensitivity and linearity of PRESAGE. Diagram (**a**) represents a relationship between the sensitivity and the linearity. (**b**) The degree of sensitivity is illustrated by the slope of the optical density vs. the absorbed dose.

The absorbed radiation dose causes the irradiated PRESAGE to change its colour. The dosimeters become darker as the absorbed dose increases. The intensity of the changes is read out in terms of optical density. The absorbed dose is directly proportional to the optical density, and the relationship between the two parameters can be indicated as dose linearity, as shown in Figure 5a. A good dosimeter should be capable of demonstrating dose linearity. The dose sensitivity of PRESAGE, on the other hand, can be illustrated by the slope of the optical density vs. the dose absorbed, as shown in Figure 5b. The steeper slope of the curve indicates a higher sensitivity of the detector to radiation. PRESAGE will exhibit different sensitivity characteristics based on the effective atomic number and the weight fraction of the leuco-dye, the free radical initiator (halocarbons), and the catalyst.

### 4.1. The Effect of Radical Initiator Concentration on the Sensitivity

The sensitivity of PRESAGE can vary upon the use of different radical initiators. The halocarbons that are often employed as radical initiators comprise various types of compounds which include iodoform, bromoform, and chloroform. Chloro-LMG and bromo-LMG have the effective atomic numbers of 7.5 and 8.14, respectively, but bromo-LMG has a higher sensitivity than chloro-LMG, which in turn has higher sensitivity than methoxy-LMG [83]. The study shows that different types and concentrations of halocarbons lead to different sensitivities. A higher effective atomic number possesses higher sensitivity. The enhancement is due to the carbon-halogen bond dissociation energy [93]. Therefore, the closest effective atomic number to water does not necessarily have optimal dosimetric properties. Thus, there is a delicate balance between the effective atomic number to retain tissue-like radiological properties and the sensitivity of the dosimeter.

A high concentration of radical initiator led to a higher effective atomic number of the PRESAGE, which was undesirable as it caused deviation from the water equivalency [80,91]. Nevertheless, the post-irradiation stability was constant for all the formulations in the study. A high radical initiator can increase the sensitivity. However, a high percentage of radical initiators can reduce the stability [75,80]. Therefore, a small concentration of radical initiator was sufficient to obtain a high sensitivity of PRESAGE with a high stability that can be maintained. The sensitivity of PRESAGE can also be enhanced by increasing the concentration of carbon tetrachloride, another radical initiator for PRESAGE, up to 30%, and the sensitivity remains the same beyond this percentage [80]. Another study reported that the ideal composition for the high sensitivity of PRESAGE is 4% LMG and 32% carbon tetrachloride [95]. The concentration of radical initiator can impact the magnitude of dose quenching in proton therapy. A study found that the concentration of radical initiator below 12% or above 18% demonstrated a rapid rise of dose quenching compared to the intermediate concentration [96]. An addition of 0.7% of dibutyltin dilaurate (DBTDL), a catalyst, also increases the sensitivity [75]. Another factor that affects the sensitivity is the volume of the dosimeter. A study shows that a large dosimeter has lower sensitivity; it is less than half the sensitivity of a small dosimeter. This is seen to be formulation-dependent and related to different hardenings of PRESAGE cured in different volumes [76]. A recent study demonstrated that dose rate influences the sensitivity of PRESAGE. The sensitivity of LMG elastomer-based PRESAGE was reduced as the dose rate increased [97]. However, the study suggests there is no dose-rate dependency if the sensitivity is observed in large number of samples.

### 4.2. The Effect of Metal Compounds on the Sensitivity

The sensitivity of PRESAGE can be enhanced further through the incorporation of metal compounds, including those which are zinc-, bismuth-, and tin-based, at a very low concentration (0.2 wt.%), without altering the radiological properties. Among the three metal-based compounds, the bismuth-based exhibited higher sensitivity [91]. These metal compounds offered advantages in accelerating polymerisation, increasing post-irradiation stability, improving post-response absorption value retention, and maintaining the dosimeter sensitivity [75,91]. Moreover, the high percentage of an organometallic catalyst can also increase the sensitivity of PRESAGE due to the bonding between the halocarbons and the metal component. A high atomic number among the organometallic compounds increases the production probability of the secondary electrons and causes the increase in radical initiator production in the halocarbons, which in turn increases the oxidation of LMG. PRESAGE (MOD) holds the closest effective atomic number to water, which is 7.416. The formulation in PRESAGE (MOD) improved post-response photostability [92]. A recent study demonstrated that PRESAGE with low Shore hardness presents lower sensitivity than PRESAGE with high Shore hardness. The study shows that the sensitivity of PRESAGE with high Shore hardness further increased by 36.6% upon incorporation with tartrazine [98]. However, PRESAGE exhibits a high effective atomic number, which compromised its water equivalency. One study incorporated zinc oxide nanoparticles and revealed a PRESAGE that has a sensitivity of 0.0105 Gy^−1^. A bromine-based RI PRESAGE was introduced recently and has improved the sensitivity of PRESAGE greatly, to 0.1109 Gy^−1^—the highest sensitivity of PRESAGE ever attained. This might be due to the addition of solvent dimethyl sulfoxide (DMSO) in the composition of PRESAGE. DMSO has a stabilising effect on the LMG that contributes to the dose response. However, its water equivalency has to be a trade-off with the effective atomic number of 9.657 [94].

A different source of ionising radiation provides a different optical absorption for PRESAGE. PRESAGE has greater sensitivity or absorption on photons when compared with the carbon ions. A study shows that the carbon ions were observed to have a lower value of the dose-response slope when compared to photons [99]. This indicates that PRESAGE has a higher sensitivity to photons than carbon ions. Table 2 summarises the sensitivity of PRESAGE based on the value of the slope or gradient. The higher the value of the slope, the higher the sensitivity.

### 4.3. Linearity of PRESAGE

The PRESAGE demonstrated a linear relationship between dose and optical response from 0 to 80 Gy, with a very good correlation coefficient of 0.9986 [101]. A study reported that the PRESAGE was capable of attaining a linearity of up to 100 Gy, with a correlation of more than 0.98. Another study found that the percentage of LMG in PRESAGE influenced its linearity. At the absorbed dose higher than 100 Gy, the PRESAGE was observed to be saturated with a lower response for another addition of doses and the relationship become non-linear. This result implied a consistent sensitivity of PRESAGE from 0 Gy to 100 Gy. In addition, the absorbance error increased for doses greater than 100 Gy [99,102]. A later investigation showed that the PRESAGE was capable of providing linearity up to 200 Gy. This result could be associated with the inclusion of DBTDL as a catalyst in the PRESAGE formulation [103].

A study demonstrated that the percentage of LMG in PRESAGE influenced the linearity. The linearity of PRESAGE was reduced (R^2^ > 0.90) upon the presence 3 wt.% of LMG. Excellent linearity (R^2^ > 0.99) was observed in the PRESAGE that had 1 wt.% and 2 wt.% LMG [80]. Another study reported that PRESAGE showed excellent linearity (R^2^ > 0.99), from 0 Gy to 30 Gy with different halocarbon radical initiators, such as iodoform, bromoform, and chloroform. The increment of bromoform and chloroform concentration increased the linearity of PRESAGE by 0.07% and 0.04%, respectively. However, the increase in the iodoform concentration reduced the linearity by 0.1% [93]. It was suggested that further research had to be conducted to look into the significant effect of halocarbon radical initiators on the linearity of PRESAGE.

The investigation of the oxygen influence on PRESAGE demonstrated that PRESAGE was capable of maintaining good linearity (R^2^ > 0.99), with or without the presence of oxygen [73,104]. Although good linearity was obtained by PRESAGE with the presence of a catalyst (DBTDL), the catalyst reduced the linearity by 2% [75]. The increment in the concentration of the catalyst could reduce the linearity. A recent study shows that the PRESAGE also retained a good linearity with the change of LMG dyes, such as MeO-LMG, Cl-LMG, and Br-LMG [83]. The various change of metal compound compositions also does not change the linearity of PRESAGE [91]. In general, over the course of the PRESAGE reformulation to obtain an optimal sensitivity, stability, and water equivalency, the linearity of PRESAGE barely deteriorated. Table 3 summarises the linearity of PRESAGE based on the correlation coefficient.

## 5. Dose Rate and Energy Dependency

### 5.1. The Dose Rate Dependency

A range of studies demonstrated that the dose response of PRESAGE was neither significantly influenced by the photon energy nor the dose rate [71,105,106]. An earlier study reported that the original PRESAGE^TM^ retained almost the same optical density (dose response) with an extremely small deviation over a different dose rate, particularly at lower doses. At 5 Gy, a difference of 2% was observed from the dose response of PRESAGE over six different dose rates. However, a notable deviation of the dose response between the different dose rates was observed at high doses of approximately 30 Gy to 50 Gy, with a difference ranging from 3% to 12% [71]. A study shows that the reusable PRESAGE, known as PRESAGE^REU^, also exhibits no dependence on the dose rate at 400 MU/min and 2400 MU/min. The study indicates that PRESAGE is capable of showing no dose rate dependency at a low dose rate and a high dose rate [107]. However, another study demonstrated that the dose response of PRESAGE was unstable at an extremely low dose rate, with an over-response rise of 16% from 0.018 Gy/min to 1.0 Gy/min. In addition, the low dose rate also led to a higher dose response [108,109]. This could be associated with the long-time exposure to the ionising radiation due to the extremely small dose rate. Thus, the chemical reaction occurred at a longer period in the PRESAGE, which yielded a high dose response. A recent study demonstrated that the incorporation of tartrazine did not influence the dose-rate dependency of PRESAGE [98]. The silicone-based radiochromic dosimeter has been developed and shows insignificant dose-rate dependency [110].

### 5.2. The Energy Dependency

The photon and electron energy showed a negligible effect on the dose response of PRESAGE [111,112]. A study found that the 5 Gy dose response of 6 MV, 10 MV, 18 MV, and 1.25 MeV showed a difference within 4% [71]. While another study reported that PRESAGE had a difference of less than 2.5% for a 5 Gy dose response from 6 MV and 18 MV [113]. Moreover, PRESAGE was also capable of producing almost the same beam profile at the different energy levels of 6 MV and 18 MV [114]. The dose response of PRESAGE showed no discrepancy between photon energy, proton energy, and electron energy [112,115]. In general, no considerable difference in terms of dose response was present within the wide range of dose rates and energy. However, the difference of dose response was clear only at the extremely low dose rates and high doses delivered to the PRESAGE. It was suggested that more research work on the energy dependence of PRESAGE be conducted to investigate the energy dependence at a high dose (>30 Gy) and an extremely low dose (<1 Gy).

## 6. Stability of PRESAGE

The stability of the dosimeter refers to the ability of the dosimeter to maintain the same dose after irradiation over time. Notably, the most stable dosimeter is the one that can maintain same dose and resistance to the fading or change of optical density for a long period of time. The original PRESAGE has a colour bleaching rate of approximately 4% per 24 h over the week. The effect of heating prior to irradiation has an insignificant effect upon the stability of PRESAGE [71].

### 6.1. The Effect of Radical Initiator

The radical initiator plays an important role in the stability of PRESAGE. A study shows that increasing the radical initiator more than 20 wt.% resulted in unstable PRESAGE over 2 days after irradiation. The colour bleaching increased to nearly 35% [80]. The higher concentration of radical initiator led to continuation of LMG oxidisation after irradiation. The advantage of a high content of radical initiator was that it increased the sensitivity of PRESAGE. However, as the radical initiator increased, the PRESAGE became more unstable. A study demonstrated that a less sensitive formulation of PRESAGE has higher stability and fading is reduced [99]. The stability of PRESAGE can be maintained by putting in a very low concentration of radical initiator. One study utilised different types of halocarbons as radical initiators and demonstrated a stable PRESAGE over a one week period after irradiation due to the small concentration of the radical initiator, which was less than 4 wt.% [93].

### 6.2. The Effect of Metal Compound

The incorporation of a small amount of metal can improve the stabilisation of PRESAGE. The incorporation of around 5% of Bi, Sn, and Zn in the formulation shows the stability of PRESAGE after more than 12 days. This is due to the metal compounds working as a singlet oxygen quencher that reduces photofading. Singlet oxygen is the main cause of the photofading when combined with leuco-dye [91,116]. In addition to the stability, the metal compounds also increase the sensitivity of PRESAGE by around 40%. However, the increase in the metal compound was limited because the high atomic numbers of the metal elements influenced the effective atomic number of PRESAGE which led to water inequivalence. One study claimed that the excessive use of an organometallic catalyst concentration increased the stability of PRESAGE. However, the high concentration of metal catalyst led to a sensitivity reduction in the PRESAGE. A small amount of tin-based catalyst has been observed to increase the stability of PRESAGE over 5 days after irradiation. Due to the small concentration of the catalyst, the sensitivity of PRESAGE remained unchanged [75]. The most recent PRESAGE, known as water-equivalence PRESAGE, has been shown to have stability up to 7 days after irradiation when stored at a low temperature (4 °C). The low temperature halted the post-processing of PRESAGE up to 100 Gy. The water-equivalence PRESAGE was unstable at a dose of 200 Gy for up to 30 min post-irradiation. However, it remained stable after that for 21 days. Despite its long stability, the water-equivalence PRESAGE is considered to have low sensitivity [103].

It can be concluded that a high concentration of radical initiator increases the sensitivity and decreases the stability of PRESAGE. Then, a high concentration of metal catalyst increases the stability and decreases the sensitivity of PRESAGE. The incorporation of metal compounds increases the stability and sensitivity of PRESAGE. However, to maintain the water equivalency of PRESAGE, there is a limitation to the amount of the metal compound. There is delicate balance between the amount of radical initiator, metal compound, and catalyst in the formulation of PRESAGE to obtain acceptable sensitivity, stability, and water equivalency.

## 7. Reusability and Reproducibility

Most PRESAGEs can typically be used once because the optical density gradually increases over time after irradiation. Following that, the absorbed dose is not reliable to read and analyse. There are a few PRESAGE dosimeters that have proven to have the potential for reusability. Instead of increased optical density, the PRESAGE gradually reduces optical density over time. After initial irradiation, the PRESAGE has the ability to return to its original optical density when exposed to room temperature.

The first reusable PRESAGE, known as PRESAGE^REU^, is capable of going through four re-irradiations. However, the PRESAGE^REU^ is only capable of reproducing a consistent absorbed dose and sensitivity during the second, third, and fourth irradiation. During the first irradiation, a lower sensitivity is present by a factor of ~2 in comparison to the consecutive re-irradiation sensitivity. The PRESAGE^REU^ requires over 12 days for optical clearing. It is believed that the newly synthesised LMG derivative plays an important role in the reusability of the PRESAGE^REU^ [117]. Another study reported that the PRESAGE-RU was effectively cleared after exposure to room temperature for 5 to 7 days in a dark room. The PRESAGE-RU shows a slight decrease in sensitivity between irradiations from the first irradiation to the fifth irradiation. However, the PRESAGE-RU only reproduced the same absorbed dose for 0 Gy to 2.5 Gy. Higher than that, the PRESAGE-RU absorbed an inconsistent dose; specifically, this occurred on the first irradiation and the fifth irradiation [118].

The most recent study demonstrated that PRESAGE could return to its original state in 2 days after irradiation. However, due to the exposure to room temperature following irradiation, the PRESAGE can only be used twice. In contrast, the PRESAGE can be reused up to four times when stored at low temperature after the irradiation. Subsequently, the results also were more stable and reliable. This study indicated that the PRESAGE in this study has high reproducibility and high reusability when stored at low temperature and only exposed to room temperature if necessary [107]. The reusability of the PRESAGE is summarised in Table 4. The percentage of reproducibility is estimated by the ratio of the same optical density linearity over the total reusability of the PRESAGE.

## 8. Readout Modalities

Magnetic resonance imaging (MRI), ultrasound, optical computed tomography (OCT), and X-ray computed tomography (X-ray CT) are the common types of systems that are employed as the readout method of the irradiated PRESAGE dosimeter. The PRESAGE exhibited an absorption peak at the wavelength of 633 nm, which is the typical absorption of oxidised leucomalachite green (LMG) [71,93,99]. Therefore, the appropriate source for optical scanning is red LED or laser of helium-neon monochromatic [93,99]. In comparison to the polymer gel and ferric gel, the strong points of PRESAGE include its significant proportion of light absorption compared to light scattering [121,122,123]. The light scattering and light refraction are able to produce artefacts in optical dosimetry. Notably, the elimination of the light-scattering impact is important in designing an ideal optical computed tomography (OCT) scanner which minimises the scattering artefact [124].

### 8.1. Magnetic Resonance Imaging (MRI)

MRI is heavily implemented as a quantification system for the Fricke gel and the polymer gel dosimeter. MRI evaluates the change of magnetic resonance which occurs in the irradiated Fricke gel dosimeter as a result of the transformation of ferrous ion (Fe^2+^) into the ferric ion (Fe^3+^). It can also assess the change of transverse and lateral relaxation time that occurs due to the polymerisation of the irradiated polymer gel. Despite being highly accurate with precise evaluation, the MRI suffers from a low signal-to-noise ratio (SNR), long scanning time, and susceptibility to imaging artefact and limited spatial resolution [18,47,125,126]. The cost of imaging a dosimeter using an MRI can be expensive [18,127].

### 8.2. Ultrasound

Ultrasound is dependent on the ultrasonic properties of the gel dosimeters, which are its acoustic velocity, ultrasound attenuation, and ultrasound flight time; these are associated with the polymerisation to read the dose absorbed by the dosimeter [60,128,129]. The ultrasound has the advantage of being low-cost and dynamic, and it can produce high-resolution images. However, the sensitivity of the readout is low, and it takes a certain period to achieve the ideal reading after irradiation [60,128]. In addition, the ultrasound is also incapable of reading the linear dose response at the lower doses (<10 Gy) [130]. Furthermore, the dose response is also observed to be saturated at a dose higher than 30 Gy, which implies its low sensitivity to reading lower doses and higher doses [60,128]. To date, no attempt at reading the dose response of PRESAGE through ultrasound has been made.

### 8.3. X-ray Computed Tomography (X-ray CT)

The X-ray CT scan, on the other hand, depends on the changes in the attenuation coefficient of the dosimeter that are characterised as the changes in electron density. The X-ray CT has the potential to become an important readout tool due to its high SNR and short scanning time. However, the major drawback of the X-ray CT is the low image contrast (thus, low-dose resolution) and low dose sensitivity [18,35,127]. In addition, the practicality of its utilisation still demands to be proven [18].

### 8.4. Optical Computed Tomography (OCT)

OCT is the most common type of system that has been used as a readout technique for the radiochromic dosimeter. The readout devices are responsible for evaluating and quantifying the dosage distribution of the dosimeter. OCT has high spatial resolution and a short scanning time and has a small physical size, which makes it portable and easy to mobilise [18,35,40]. Furthermore, it also provides high SNR due to the laser beam high intensity and the capability to scan large samples without the necessity of high-cost optical components [35,81]. The first generation of OCT, commercialised as OCTOPUS, has been the only commercial OCT for a number of years. The first generation of OCT capable of producing high quality images was pointed out as a “gold standard” [131]. Even more, it has the ability to remove light contamination [40]. A slow scanning speed is the major drawback of the laser-scanned OCT; the speed is 12 min per slice with 128 × 128 pixels [132]. OCTOPUS has improved the scanning time to 5 min per slice. However, full 3D imaging still requires up to 16 h [133].

The second generation of OCT was developed as an alternative for a faster scanner that is based on a coupled-charged detector (CCD) and a broad cone light beam that is commercially known as Vista [132,134]. The CCD-based OCT provides an advantage over its first generation in terms of scanning speed due to the CCD chip. It delivers complete two-dimensional (2D) projection in an instant instead of forming 2D distribution from collected one-dimensional projection, which significantly consumes time. The CCD-based OCT is capable of obtaining a complete 2D image as fast as the laser-scanned OCT obtains a 1D image [132]. The broad scanned light gives advantages when scanning a big sample, without the necessity of purchasing expensive optical component. It is cheap and easily scalable [135]. However, it suffers from a scattered radiation issue that needs various methods of correction to reduce the scattered light artefacts [136]. The parallel beam scanner with a telecentric lens is another type of CCD-based OCT that has the advantage of reducing the scattered radiation effect [137].

**Figure 6:**
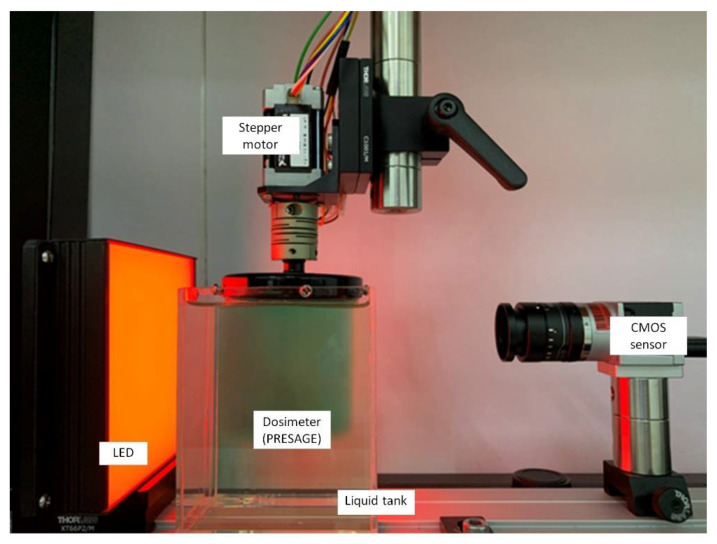
The in-house CMOS-OCT dosimetry system from the Laboratory of Medical Physics & Simulation, Universiti Teknologi MARA. The system primarily consists of CMOS sensor, the dosimeter, LED, and stepper motor.

Another OCT system was developed based on the Complementary Metal Oxide Semiconductor (CMOS) active pixel image sensor. A CMOS image sensor has higher signal-to-noise ratio (SNR), higher spatial resolution, and a faster frame rate when compared to the CCD chip. The active pixel has the ability to integrate signal processing at the pixel level. The CMOS has gained increasing attention as a competitive technology to CCD and has been used in high-end consumer products and scientific instruments [35,138]. A CMOS-OCT system has been developed as a measurement in radiotherapy [35,138]. A study demonstrated that a CMOS-OCT was able to produce a multidimensional dose analysis with consistent results [139]. The CMOS-OCT system was also capable of visualising stereotactic radiosurgery (SRS) treatment dose distribution in a PRESAGE dosimeter. The system can measure dosimetric and geometric information during radiotherapy delivery accurately. In addition, the CMOS-OCT system also provides a shorter scanning time than the CCD-based OCT, with a higher dynamic range [140,141]. Figure 6 shows the CMOS-OCT imaging system, which consists of a CMOS sensor, stepper motor, LED, and dosimeter.

## 9. Dosimetry Applications

### 9.1. Applications in Radiotherapy Dosimetry

Advances in radiotherapy have allowed the conformal delivery of a high radiation dose to a cancer target volume whilst sparing the dose to normal tissues through various IMRT techniques [142,143]. PRESAGE provides dose distribution in 3D that can be compared with the dose predicted from the radiotherapy treatment planning system (TPS). Gamma analysis is used to compare the delivered dose distribution to that planned by the TPS. The calculation is based on the distance to agreement (DTA) criteria and the dose difference criteria [144]. Recent studies show that PRESAGE has excellent gamma passing rates for various radiotherapy treatments [145,146,147,148,149,150,151,152,153].

PRESAGE was used with the Radiological Physics Center head and neck (RPC H&N) phantom for IMRT verification and was shown to have excellent intra-dosimeter consistency within 2%. The dosimeter is capable of producing a consistent response (by a difference within 2%) at three different 3D inserts. The PRESAGE also showed a gamma passing rate of more than 99% for 2%/2 mm criteria when the 4 mm ring profile around the edge was removed from the analysis. The presence of impurities caused errors at the particular points and was responsible for 1% of the failures [123]. Furthermore, the study also showed that the dosimeter measurement agreed well with the EBT film (98% pass rate) and the Eclipse dose calculations (94% pass rate) [154]. The dosimeter has post-irradiation stability for more than 90 h.

PRESAGE has also been shaped into an anthropomorphic breast for IMRT treatment verification and brachytherapy [155,156]. The study demonstrated that the dose measured by PRESAGE was within a 5% maximum difference when compared with the EBT2 film and the Pinnacle treatment planning system. The gamma passing when compared with the EBT2 film and Pinnacle TPS was 88.4% and 90.6%, respectively, at the 3%/3 mm criteria. The major failures took place at the 8 mm outer ring of the dosimeter. PRESAGE illustrated a 95% gamma passing rate if the ring was ignored [156]. PRESAGE has also been fabricated as a sheet and used for QA measurement. A study shows that the PRESAGE sheet is capable of producing a linear dose response with a negligible dose rate and energy dependence. It demonstrated a gamma passing rate of 99.7% when compared with the EBT3 films [113]. Another study shows that the PRESAGE sheet is capable of being reused six times after irradiation, with a consistent sensitivity within 5% [157]. These works have provided the applicability of PRESAGE as a fashionable phantom. The investigation of the feasibility of the PRESAGE breast phantom for other radiotherapy treatments was suggested.

Another study has developed a new formulation of PRESAGE, known as the DEA-1 formulation, to investigate its feasibility for IMRT and VMAT treatment verification. The study reported that PRESAGE shows deviation within 2% for VMAT and less than 2% for IMRT, as compared with the Eclipse calculations in the high-dose regions. The study also showed that the PRESAGE has average gamma passing rates of more than 98% for IMRT and around 92% for VMAT, when compared to the Eclipse calculations. Similar to the previous studies, the outer ring of the dosimeter was the major failure in the gamma passing rate and was left out in the gamma analysis [76].

PRESAGE has been utilised to investigate the effect of organ motion on IMRT treatment and shows a good agreement of more than 90% passing rates for 3%/2 mm criteria, with the noise being less than 0.5%. A significant deviation was observed in the form of stretching and shifting for organ motion treatment. [158]. A study showed that PRESAGE has a gamma passing rate of 98% for 3%/2 mm criteria in VMAT treatment [159]. PRESAGE has also been utilised in an RPC head and neck phantom for 3D-CRT and VMAT treatment, with the gamma passing rate of 99% when compared to the Pinnacle calculations for 5%/3 mm criteria [160].

PRESAGE has been used for the Imaging and Radiation Oncology Core (IROC) Houston Quality Assurance Center (IROC) head and neck phantom as a QA tool for VMAT treatment. The study demonstrated the feasibility of the dosimeter with gamma passing rates of 94.38% for 5%/3 mm [147]. One study reported that the gamma passing rates of PRESAGE were 99% for 3%/3 mm criteria when investigating the applicability of the dosimeter for organ motion in VMAT treatment. However, the difference of 15% in dose to PTV was observed [161]. In general, PRESAGE demonstrated useful properties when verifying several radiotherapy methods, including IMRT and VMAT.

### 9.2. The Challenge in Small Field Dosimetry

**Figure 7:**
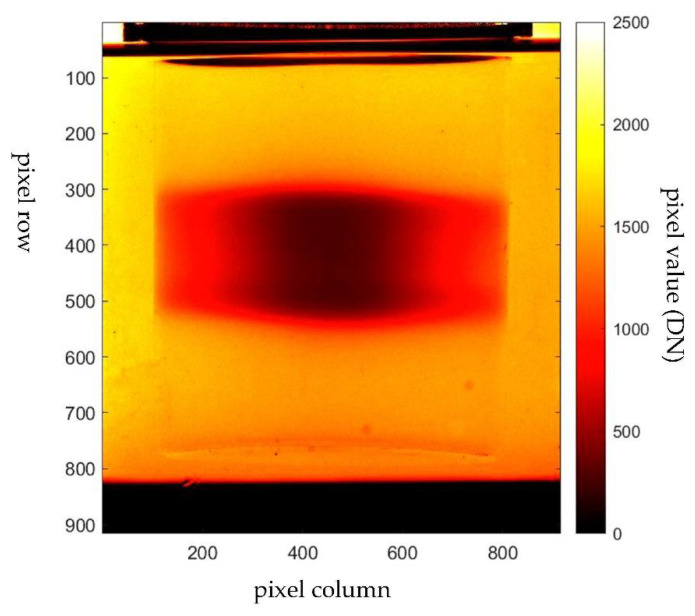
Projection image of PRESAGE irradiated with stereotactic radiosurgery (SRS) treatment. The image was captured with the in-house CMOS-OCT dosimetry system.

Another challenge in radiotherapy dosimetry is the treatment of a small volume of cancer target. The small field treatment uses a few mm beam apertures to irradiate a small volumetric target. A recent study shows that the PRESAGE is capable of obtaining a small-field megavoltage beam accurately [152]. PRESAGE has been used to make a measurement of a small field as small as 5 mm^2^. A study shows that PRESAGE has an accuracy of 99.8% at a field size of 20 mm^2^. The accuracy, however, is reduced as the field size is reduced. At 5 mm^2^, the accuracy of PRESAGE is 96.4%. When compared with the EBT film, PRESAGE has better accuracy at the large field size. At the small field size, the EBT film was observed to have better accuracy. This is due to the debris and small bubbles suspended in the PRESAGE matrix. In addition, the error also included the water inequivalence that may raise the equilibrium of the lateral electronics a little and affect the measured scatter factors [111,162]. Such errors can be improved through the development of PRESAGE formulation. The utilisation of PRESAGE in gamma knife radiosurgery has also been investigated. The study shows that PRESAGE has excellent agreement with the gamma knife output factors, with the average difference of 1.24%, which is suitable for performing quality assurance measurements for the radiosurgery gamma knife treatment system [163]. Figure 7 shows an example of an SRS dose deposited in a PRESAGE dosimeter.

### 9.3. Application in Brachytherapy

PRESAGE has also been used in high-dose brachytherapy treatment. A current benchmark for the measurement of the absolute dose in brachytherapy is TLD. The main disadvantage of TLD is water inequivalence. The dose response of TLD depends on dose rate and beam energy. Thus, 3D dosimetry is required to enhance the accuracy and precision of absolute dose measurement in brachytherapy [164,165]. PRESAGE shows more accurate absolute dose measurement when compared with the TLD in brachytherapy treatment. A study shows that PRESAGE has a deviation of 0.7% from the treatment dose prediction. Meanwhile, the TLD has a deviation of 13.08%. In addition, PRESAGE also has a 98.9% agreement with the EBT2 film dose delivery in brachytherapy treatment [166]. Another study reported that PRESAGE provides an acceptable relative dose measurement for Ir-192 and Cs-137 brachytherapy sources [167]. PRESAGE has also demonstrated feasibility in the application to the anthropomorphic breast shape for measurement of the skin dose for accuracy verification in the brachytherapy treatment planning system [168].

## 10. Conclusions

The PRESAGE polymer dosimeter has emerged to overcome the disadvantages of the previous three-dimensional dosimeters, the Fricke gel and the polymer gel dosimeter. It has displayed several advantages over the polymer gel dosimeter, such as its ability to be fabricated in any variety of shape without any vessel due to its being solid state; it does not manifest diffusion; it is highly stable and insensitive to oxygen contamination; it has better optical evaluation and can be formulated to any dose range that is applicable to radiotherapy. Furthermore, the presence of oxygen can enhance the PRESAGE sensitivity during fabrication. Additionally, the optical response as a result of light attenuation with a minimum perturbation of light scattering has provided a considerable advantage to the polymer-based dosimeter over the polymer gel dosimeter.

For more than a decade, many efforts have been made for the advancement in PRESAGE development in terms of its stability, sensitivity, water equivalency, and feasibility in clinical applications. The improvement of the sensitivity of PRESAGE was achieved by incorporating metal compounds, diversifying the concentration of the radical initiators, changing the derivatives of LMG, and changing the catalyst. The challenge in improving the sensitivity is to make a careful formulation to ensure that PRESAGE possesses acceptable water equivalency, whether in high-dose or low-dose radiation. Furthermore, the concentration level of the radical initiator, metal catalyst, and metal compounds impacts the PRESAGE stability. Metal compounds can lead to higher sensitivity and stability of PRESAGE. However, there is a limitation to the amount of the metal compound needed to maintain the water equivalency of PRESAGE. The mixture requires the maintaining of a delicate balance between the amount of radical initiator, the metal compound, and the catalyst in the formulation of PRESAGE to obtain acceptable sensitivity, stability, and water equivalency. Currently, the PRESAGE shows a close equivalence to water at higher energy. At the lower energy, however, the PRESAGE has yet to retain water equivalence. Therefore, there is a need for improvement in this area.

In conclusion, the implementation of PRESAGE dosimetry is useful for complex radiotherapy treatment verifications, which include VMAT, IMRT, radiosurgery, and brachytherapy. The dosimeter also has great potential for other radiotherapy treatment techniques, including Stereotactic Ablative Radiation Therapy (SABR), Stereotactic Body Radiation Therapy (SBRT), proton beam therapy, and Intraoperative Radiotherapy (IORT). In addition, the PRESAGE is also promising for the dosimetry audit of advanced radiotherapy treatment. It is suggested that further research work regarding the irradiation and readout is performed for the application of PRESAGE in radiotherapy audit practices.

## Figures and Tables

**Table 1:** The low-energy and high-energy cross-section of PRESAGE.

Material	$\rho_{e}/\rho$	Effective Atomic Number (Z_eff_)	Low-Energy Cross-Section Deviation from Water	High Energy Cross-Section Deviation from Water
Water	3.3428	7.417	-	-
PRESAGE (original) [72]	3.2826	8.650	81%	<5%
PRESAGE (Formulation A) [84]	3.2669	7.688	40%	3%
PRESAGE (Formulation B) [84]	3.2754	7.740	49%	4%
PRESAGE (Formulation C) [84]	3.2670	8.652	117%	2%
PRESAGE (Iodoform) [93]	3.2611	16.03	96%	<4%
PRESAGE (MOD3) [92]	3.2768	7.416	<18%	N/A (small)
PRESAGE (DBTDL) [90]	N/A	N/A	2%	N/A (small)
PRESAGE (Bromine-based RI, F2) [94]	3.4420	7.425	8%	N/A (small)
PRESAGE (Bromine-based RI, F5) [94]	3.6580	9.657	>50%	N/A (small)

**Table 2:** The sensitivity of various PRESAGEs with their corresponding density and effective atomic number.

Material	Density (g/cm^3^)	Effective Atomic Number (Z_eff_)	Slope Value (Sensitivity, Gy^−1^)
PRESAGE (LMG) [100]	1.048	7.45	0.00890
PRESAGE (MeO-LMG) [100]	1.052	7.46	0.01802
PRESAGE (Cl-LMG) [100]	1.054	7.50	0.03027
PRESAGE (Br-LMG) [100]	1.057	8.10	0.04018
PRESAGE (Bi Neo) [91]	1.085	7.90	0.00787
PRESAGE (DBTDL) [91]	1.084	7.65	0.00681
PRESAGE (Zn OCT) [91]	1.083	7.49	0.00658
PRESAGE (Iodoform) [93]	1.047	16.03	0.02333
PRESAGE (Bromoform) [93]	1.076	9.96	0.01616
PRESAGE (Chloroform) [93]	1.102	6.62	0.00570
PRESAGE (Organometallic catalyst) [75]	1.100	7.72	0.01400
PRESAGE (MOD 1) [92]	1.039	7.410	0.00557
PRESAGE (MOD 2) [92]	1.042	7.415	0.00646
PRESAGE (MOD 3) [92]	1.044	7.416	0.00722
PRESAGE (tartrazine) [98]	N/A	11.100	0.10100
PRESAGE (bromine-based RI, F5) [94]	1.135	9.657	0.11090
PRESAGE (bromine-based RI, F2) [94]	1.058	7.380	0.02440

**Table 3:** The correlation coefficient that represents degree of linearity for various PRESAGEs.

Material	Dose Range	Correlation Coefficient
PRESAGE (LMG) [100]	0 Gy–30 Gy	0.9946
PRESAGE (MeO-LMG) [100]	0 Gy–30 Gy	0.9963
PRESAGE (Cl-LMG) [100]	0 Gy–30 Gy	0.9985
PRESAGE (Br-LMG) [100]	0 Gy–30 Gy	0.9997
PRESAGE (Bi Neo) [91]	0 Gy–30 Gy	0.9999
PRESAGE (DBTDL) [91]	0 Gy–30 Gy	0.9999
PRESAGE (Zn OCT) [91]	0 Gy–30 Gy	0.9999
PRESAGE (Iodoform) [93]	0 Gy–30 Gy	0.9988
PRESAGE (Bromoform) [93]	0 Gy–30 Gy	0.9996
PRESAGE (Chloroform) [93]	0 Gy–30 Gy	0.9980
PRESAGE (Organometallic catalyst) [75]	0 Gy–20 Gy	0.9700
PRESAGE (MOD 1) [92]	0 Gy–50 Gy	0.9984
PRESAGE (MOD 2) [92]	0 Gy–50 Gy	0.9993
PRESAGE (MOD 3) [92]	0 Gy–50 Gy	0.9989
PRESAGE (deoxygenation) [104]	0 Gy–30 Gy	0.9981
PRESAGE (no deoxygenation) [104]	0 Gy–30 Gy	0.9972

**Table 4:** Reusability and reproducibility of PRESAGE.

Material	Rate of Optical Clearing	Reusability	Reproducibility
PRESAGE^REU^ [117]	>12 days	5 times	80%
PRESAGE^®^ [119]	14 days	N/A	N/A
PRESAGE-RU [118]	5–7 days	5 times	60%
PRESAGE-RU [120]	10 days	3 times	66%
PRESAGE^REU^ (room temperature) [107]	2 days	2 times	50%
PRESAGE^REU^ (low temperature) [107]	2 days	3 times	75%

## Data Availability

Not applicable.
